# Refractive results of photorefractive keratectomy comparing trans-PRK and PTK−PRK for correction of myopia and myopic astigmatism

**DOI:** 10.1007/s10792-024-02999-w

**Published:** 2024-02-25

**Authors:** Ahmed Saad, Amr Saad, Andreas Frings

**Affiliations:** 1 Department of Ophthalmology, Heinrich Heine University, Duesseldorf, Germany; 2 Augenlaser−Zentrum Kärnten, Klagenfurt am Wörthersee, Klagenfurt, Austria; 3 Augenheilkunde and Augenlaser Zentrum PD Dr. med. A. Frings, Nuremberg, Germany

**Keywords:** Refractive surgery, Photorefractive keratectomy, Phototherapeutic keratectomy, Myopia

## Abstract

**Purpose:**

To compare refractive outcomes after transepithelial photorefractive keratectomy (*t*PRK) and combined phototherapeutic keratectomy (PTK−PRK) procedure using two different excimer laser platforms for correction of myopia and myopic astigmatism.

**Methods:**

In this retrospective multicenter study, we compared the results of two different PRK methods. The first group received a *t*PRK treatment with the Amaris750 excimer laser (Schwind eye-tech solutions). The second group received a combined PTK−PRK treatment with the MEL90 excimer laser (Carl Zeiss). Only healthy eyes with no previous surgery and a spherical equivalent (SE) of −1 to −8 diopters (*D*) were included. Preoperative spherical equivalent (SE), age, and sex were matched among the two groups. All treatments were performed by the same surgeon in different clinics. This study was approved by the local Ethics Committee (No. 2022–1980).

**Results:**

We included 154 eyes of 86 patients in our study. There was no difference in predictability of SE between the two groups. Efficacy and safety indices were equally high in both groups. Similarly, no significant differences were seen in change of higher order aberrations (HOA) between the two groups (*p* > 0.05). No complications occurred.

**Conclusion:**

Both investigated methods provide safe and effective refractive results. The combination of PTK with PRK may be a suitable option to the already used one-step *t*PRK for the correction of myopia.

## Introduction

Refractive surgery is one of the most commonly performed procedures in medicine. The field of refractive surgery has evolved rapidly in recent years. Several surgical treatment options are now available, including photorefractive keratectomy (PRK), laser in situ keratomileusis (LASIK), and small incision lenticule extraction (SMILE). Nevertheless, PRK remains a popular method for correcting refractive errors, especially in eyes with thin corneas, high refractive errors, or recurrent corneal erosions [1, 2].

PRK can be performed in different ways. Either as a single-step procedure, in which the corneal epithelium and stroma are ablated simultaneously with an excimer laser, called transepithelial PRK (tPRK), or as a two-stage procedure, in which it is possible to distinguish between the mechanical, alcohol-assisted, or laser-assisted method of epithelial ablation. The introduction of *t*PRK in the 1990s aimed to avoid complications associated with epithelial debridement [3] and showed at least non-inferior results compared to two-step PRK [4]. However, there are only a few devices currently available in the market for the single-step PRK (e.g., SCHWIND Amaris and Alcon Wave Light). Representative of this procedure is the Amaris750 excimer laser. Therefore, the two-step method is still used by surgeons who do not have access to these laser platforms.

Basically, the two-stage procedure, consisting of mechanical epithelial and subsequent stromal ablation, is used in PRK treatment with the MEL90 excimer laser platform (Carl Zeiss Meditec). Excimer lasers like this are also used for PTK in other indications (e.g., degenerative diseases of the cornea) [5, 6]. Therefore, the sequential combination of PTK followed by PRK represents an option for non-contact laser correction with the MEL90.

On the one hand, the *t*PRK method has been proven many times as a safe and effective way to treat myopia [7]. On the other hand, the PTK−PRK procedure seems to show stable results, as demonstrated previously by two groups [8, 9] with the EX500 laser (Alcon Laboratories). Also, older publications indicate that the PTK−PRK combination is an effective treatment [10, 11]. However, only one of the previous works compared the PTK−PRK method with the *t*PRK and none of them evaluated the two-stage PTK−PRK method using the MEL90 excimer laser. This retrospective data analysis aims to compare these two approaches (*t*PRK vs. PTK−PRK) in terms of postoperative outcomes. Here, the standard target parameters of visual acuity, efficacy, and safety indices are considered and analyzed [12]. Hereby, we want to make an important contribution to this novel modified PRK application with the MEL90 and offer users of this excimer laser more evidence for individual therapy decisions. It is interesting to ask whether these two methods differ significantly. To the best of our knowledge, there is very little work on PTK−PRK procedures using MEL90, especially in the field of refractive surgery. Thus, further research on this issue is necessary to provide proper evidence for this alternative method.

## Methods

In this retrospective study, we compare data from 154 eyes of 86 patients, treated at the same private practices. This study was approved by the local research ethics committee (No. 2022–1980) and complies with the Declaration of Helsinki. Our study is registered in the German Clinical Trials Register, DRKS (DRKS−ID: DRKS00030977). All patients gave informed consent for the use of their routinely collected data for research purposes. In addition, all patients were older than 18 years and none had any ocular disease, previous ocular surgery or trauma, or general disorders affecting the eye. Patients with any systemic diseases that might affect the eye were excluded from surgery. We included only eyes with a spherical equivalent (SE) of −1 to −8 diopters (*D*).

For comparison, we formed two groups. Group A patients received conventional *t*PRK treatment with Amaris750. Group B patients underwent refractive surgery using the combined PTK−PRK method using the MEL90 laser. To avoid sampling bias, we randomly select the 154 eyes (86 patients) from a larger cohort (approximately 197 eyes) using the random number function of Excel (Microsoft Excel 2017, Microsoft®). To reduce any bias caused by different preoperative refractive errors and thus different ablation depths, all groups were matched regarding the preoperative SE.

All patients were examined before surgery according to a standard protocol. Visual acuity in terms of subjective refraction, including uncorrected distance visual acuity (UDVA) and corrected distance visual acuity (CDVA), was collected within 2 weeks before and 6 months after surgery. We also obtained topographic data using Schwind Anterior Segment Analyzers (Peramis and Sirius, Schwind eye−tech solutions) for *t*PRK patients and the TMS−5 Scheimpflug tomograph (Tomey) and WASCA aberrometer (Carl Zeiss) for PTK−PRK patients all at an optical zone (OZ) of 6.5 mm. For treatment planning, we used the *K* values of the respective topography device as well as those of an auto-refractometer/keratometer (Nidek).

All operations were performed by the same surgeon using two different laser platforms with a repetition rate of 500-Hz for the MEL90 and 750-Hz for the Amaris 750. For all treatments, topical anesthesia (Oxybuprocaine, Conjuncain EDO® 0.4 mg/mL, Bausch & Lomb) was used. Patients were asked to focus their gaze on the fixation light to center the ablation zone. Group A patients underwent a single stage tPRK procedure in which the excimer laser simultaneously reshapes the corneal epithelium and stroma to correct the refractive error. Patients in group *B* first received PTK treatment for epithelial ablation, with a depth of 50 μm and diameter of 8mm. Following this, refractive ablation of the stroma was performed using the PRK mode, without any further modifications. Our own ablation nomogram was utilized for this step, and no wavefront-guided mode was employed. It is important to acknowledge that the SCHWIND Amaris750 incorporates an aspherical epithelium ablation, taking into consideration the impact of keratometry and the associated higher energy loss in the periphery. In contrast, the MEL−90 does not account for peripheral energy loss. Regardless of the method employed, all refractive ablations were consistently performed with an OZ of 6.5 mm and a transition zone of 1.5 mm. Mitomycin C (MMC, 0.02%) was applied for 15–30 s (sec), depending on ablation depth. For corrections up to −4 *D*, MMC was applied for 20 s, while durations of 45 s were employed for corrections exceeding −4 *D*.

At the end of the procedure, all eyes got a therapeutic contact lens for 5 days, as well as preservative-free eyedrops of ofloxacin (Floxal EDO®, Bausch & Lomb) and dexamethasone (Dexa EDO®, Bausch & Lomb). Postoperative care included the application of Nepafenac eyedrops (0.1%, Nevanac®, Novartis) for 5 days, as well as hyaluronan (Hylo−Comod®, Ursapharm) eyedrops and dexamethasone (Dexa EDO®, Bausch & Lomb) eyedrops for 6 weeks.

Statistical analysis was performed using R Core Team software (R Foundation for Statistical Computing 2021). Refractive results are represented as standard graphs for reporting outcomes in refractive surgery [12], which show the efficacy, safety, predictability, and accuracy of each treatment. The efficacy index (EI) describes the ratio between postoperative UDVA and preoperative CDVA, whereas the safety index (SI) describes the ratio between postoperative CDVA and preoperative CDVA. Predictability is evaluated as the proportion of eyes achieving a postoperative SE within ± 0.50 *D* of targeted visual acuity and was analyzed using the least squares method. The differences in percent (%) of eyes within 0.50 *D* between the groups were tested with Fisher's exact test.

The differences in pre- and postoperative parameters were tested using either the independent *t*−test or Mann–Whitney test, based on whether the assumptions of parametric test were satisfied. Normality was tested with Shapiro–Wilk test, homogeneity of variances with Levene test, and outliers, if any, were identified using the box plot method. The changes within the groups were tested with either Wilcoxon signed rank test or paired *t* test. And the differences in changes were tested with either Mann–Whitney test or independent *t* test.

## Results

In this study, we evaluated the refractive outcomes of 154 eyes of 86 patients. Both treatment groups (*t*PRK and PTK−PRK) were matched in terms of preoperative refraction, age, and sex to achieve more accurate analysis. The mean age in both treatment groups was 35 years. No complications occurred postoperatively.

Table 1 compares the preoperative descriptive data of the two groups and the total cohort. In addition to SE values, coma, trefoil, and spherical aberration (SA) and higher order aberrations (HOA) are also listed. There is no significant difference between the two groups regarding all parameters (*p* > 0.05). Table 2 shows the same parameters 6 months postoperatively for the two groups and the complete cohort. Again, there is no significant difference between the two groups (*p* > 0.05). Table 3 summarizes the differences in pre- and postoperative parameters within the two groups and between the groups, respectively. The postoperative change in sphere, cylinder, and SE was highly significant in the two groups (*p* < 0.001). However, the differences in changes between the two groups were not statistically significant in all other parameters.

**Table 1:** Preoperative descriptive data (subjective refraction and higher order aberrations)

Parameter	*t*PRK (*N* = 77)	PTK−PRK (*N* = 77)	Total (*N* = 154)	*p* value
Manifest sphere (*D*)				*W* = 2995, *p* = 0.913, *r* = − 0.009^a^
Range	− 1.25, −7.50	− 1.50, −7.50	− 1.25, −7.50	
Mean (SD)	− 4.31 (1.77)	− 4.29 (1.75)	− 4.30 (1.76)	
Median (*Q*1, *Q*3)	−4.00 (−2.75, −6.25)	−4.25 (−3.00, −6.00)	−4.00 (−3.00, −6.00)	
Manifest cylinder (*D*)				*W* = 2908, *p* = 0.836, *r* = −0.017^a^
Range	−1.75, 0.00	−1.75, 0.00	−1.75, 0.00	
Mean (SD)	−0.45 (0.41)	−0.45 (0.45)	−0.45 (0.43)	
Median (*Q*1, *Q*3)	−0.25 (−0.75, −0.20)	−0.25 (−0.75, 0.00)	−0.25 (−0.75, 0.00)	
Manifest spherical equivalent (*D*)				*W* = 2984.5, *p* = 0.944, *r* = −0.006^a^
Range	−1.00, −7.38	−1.25, −7.00	−1.00, −7.38	
Mean (SD)	−4.09 (1.81)	−4.06 (1.78)	−4.08 (1.79)	
Median (*Q*1, *Q*3)	−3.88 (−2.62, −5.88)	−4.00 (−2.75, −5.88)	−3.88 (−2.62, −5.88)	
Coma				W = 2867, *p* = 0.726, *r* = −0.028^a^
Range	0.01, 0.47	0.00, 0.51	0.00, 0.51	
Mean (SD)	0.11 (0.07)	0.12 (0.08)	0.11 (0.07)	
Median (*Q*1, *Q*3)	0.10 (0.06, 0.14)	0.11 (0.06, 0.15)	0.11 (0.06, 0.15)	
Tefoil				W = 2601, *p* = 0.189, *r* = −0.106^a^
Range	0.01, 0.28	0.01, 0.28	0.01, 0.28	
Mean (SD)	0.11 (0.07)	0.12 (0.06)	0.11 (0.06)	
Median (*Q*1, *Q*3)	0.09 (0.06, 0.14)	0.11 (0.08, 0.15)	0.10 (0.06, 0.15)	
Spherical aberration				*t* (150.9) = 0.11, *p* = 0.916, *r* = 0.009^b^
Range	−0.13, 0.18	−0.11, 0.21	−0.13, 0.21	
Mean (SD)	0.07 (0.06)	0.07 (0.06)	0.07 (0.06)	
Median (*Q*1, *Q*3)	0.08 (0.02, 0.12)	0.07 (0.02, 0.10)	0.07 (0.02, 0.11)	
Higher order aberration				W = 2783, *p* = 0.513, *r* = −0.053^a^
Range	0.11, 0.65	0.11, 0.72	0.11, 0.72	
Mean (SD)	0.23 (0.08)	0.24 (0.09)	0.23 (0.09)	
Median (*Q*1, *Q*3)	0.21 (0.18, 0.27)	0.22 (0.18, 0.29)	0.21 (0.18, 0.28)	

^a^ Mann–Whitney test, ^b^ Independent *t* test, *D* diopter, *SD* standard deviation, *Q*1 first quartile, *Q*3 third quartile

**Table 2:** Postoperative descriptive data (subjective refraction and higher order aberrations)

Parameter	*t*PRK (*N* = 77)	PTK−PRK (*N* = 77)	Total (*N* = 154)	*p* value
Manifest sphere (*D*)				*W* = 3201.5, *p* < 0.001*, r* = −0.287^a^
Range	−0.50, 0.25	−0.50, 0.25	−0.50, 0.25	
Mean (SD)	−0.08 (0.18)	−0.19 (0.20)	−0.14 (0.19)	
Median (*Q*1, *Q*3)	0.00 (−0.25, 0.00)	−0.25 (−0.25, 0.00)	0.00 (−0.25, 0.00)	
Manifest cylinder (*D*)				*W* = 2490.5, *p* = 0.376, *r* = −0.076^a^
Range	−0.25, 0.00	−0.50, 0.00	−0.50, 0.00	
Mean (SD)	−0.11 (0.13)	−0.14 (0.16)	−0.13 (0.14)	
Median (*Q*1, *Q*3)	0.00 (−0.25, 0.00)	0.00 (−0.25, 0.00)	0.00 (−0.25, 0.00)	
Manifest spherical Equivalent (*D*)				*W* = 3202.5*, p* < 0.001*, r* = −0.340^a^
Range	−0.50, 0.25	−0.62, 0.25	−0.62, 0.25	
Mean (SD)	−0.13 (0.16)	−0.26 (0.19)	−0.20 (0.19)	
Median (*Q*1, *Q*3)	−0.12 (−0.25, −0.06)	−0.25 (−0.38, −0.12)	−0.25 (−0.25, −0.12)	
Coma				*W* = 2631, *p* = 0.897, *r* = −0.011^a^
Range	0.00, 0.45	0.02, 0.53	0.00, 0.53	
Mean (SD)	0.13 (0.08)	0.14 (0.09)	0.14 (0.08)	
Median (*Q*1, *Q*3)	0.12 (0.08, 0.18)	0.12 (0.08, 0.18)	0.12 (0.08, 0.18)	
Tefoil				*W* = 2536, *p* = 0.616, *r* = −0.042^a^
Range	0.02, 0.28	0.02, 0.35	0.02, 0.35	
Mean (SD)	0.10 (0.06)	0.10 (0.06)	0.10 (0.06)	
Median (*Q*1, *Q*3)	0.09 (0.05, 0.13)	0.09 (0.06, 0.14)	0.09 (0.06, 0.14)	
Spherical aberration				*t* (143.4) = −0.06, *p* = 0.949, *r* = 0.005^b^
Range	−0.15, 0.22	−0.17, 0.23	−0.17, 0.23	
Mean (SD)	0.04 (0.08)	0.05 (0.08)	0.05 (0.08)	
Median (*Q*1, *Q*3)	0.04 (−0.01, 0.10)	0.04 (0.00, 0.10)	0.04 (−0.01, 0.10)	
Higher order aberration				*W* = 2380, *p* = 0.266, *r* = −0.092^a^
Range	0.12, 0.57	0.11, 0.52	0.11, 0.57	
Mean (SD)	0.24 (0.08)	0.26 (0.09)	0.25 (0.09)	
Median (*Q*1, *Q*3)	0.23 (0.19, 0.27)	0.23 (0.19, 0.32)	0.23 (0.19, 0.29)	

^a^ Mann–Whitney test, ^b^ Independent *t* test, *D* diopter, *SD* standard deviation, *Q*1 first quartile, *Q*3 third quartile

**Table 3:** Tests for changes after treatment

	*t*PTK^†^	PTK−PRK^*^	PTK−PRK vs. tPTK^‡^
Manifest sphere (*D*)	< 0.001^b*^	< 0.001^b*^	0.848^c^
Manifest cylinder (*D*)	< 0.001^b*^	< 0.001^b*^	0.174^c^
Manifest spherical equivalent (*D*)	< 0.001^b*^	< 0.001^b*^	0.953^c^
Coma	0.080^b^	0.038^a^	0.681^c^
Tefoil	0.087^a^	0.247^a^	0.662^d^
Spherical aberration	0.021^b^	0.029^b^	0.950^c^
Higher order aberration	0.140^a^	0.385^a^	0.521^d^

*a* Paired *t* test, *b* Wilcoxon signed rank test, *c* Mann–Whitney test, *d* Two−sample *t* test, * Tests changes within PTK−PRK, † Tests changes within *t*PTK, ‡ Tests differences in changes between PTK−PRK and *t*PTK

**Fig. 1:**
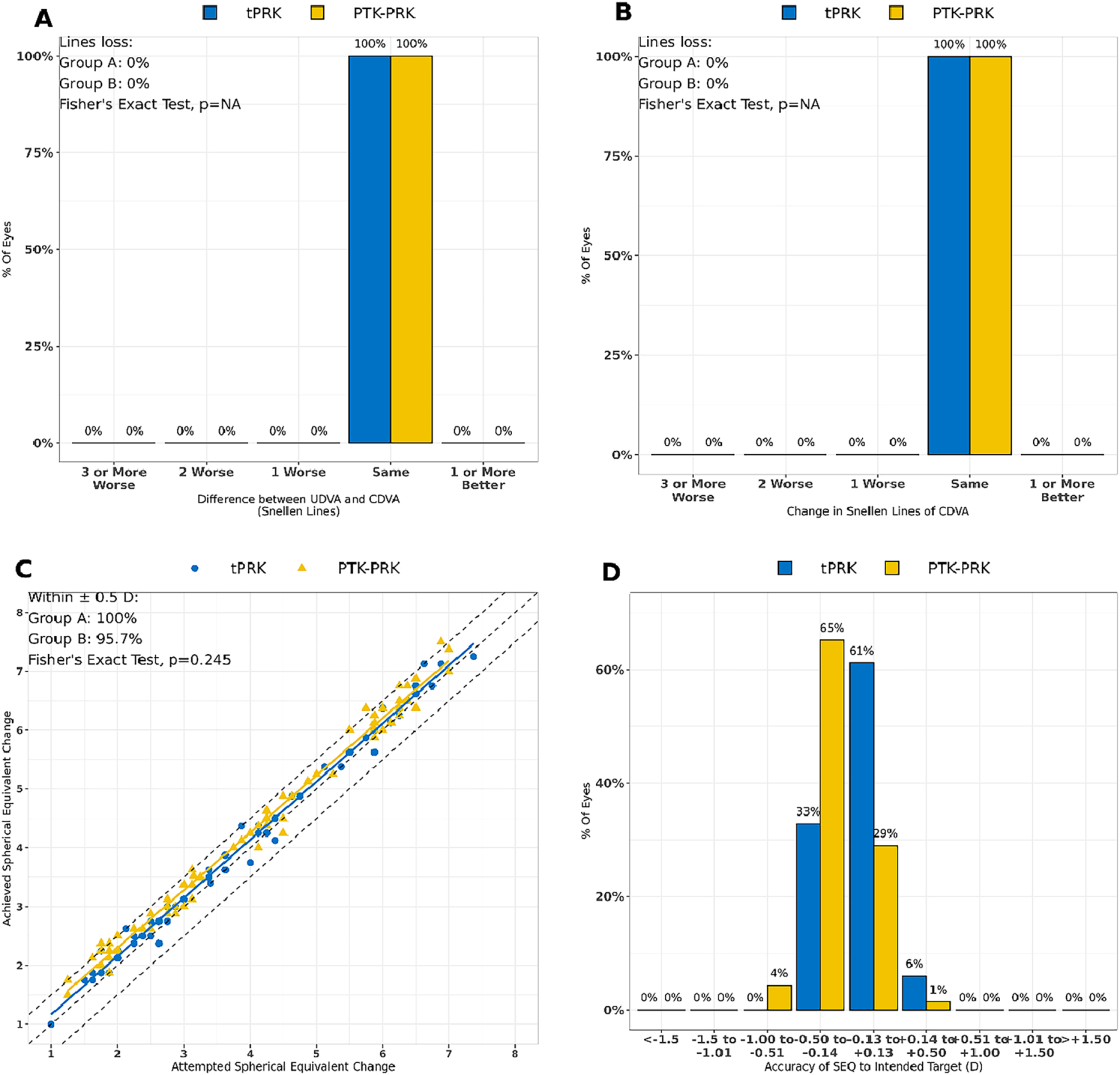
Standard graphs reporting visual outcomes for Groups A and B: **A** Efficacy graph, **B** Safety graph, **C** Precision graph, **D** Accuracy graph; *t*PRK: transepithelial photorefractive keratectomy, PTK−PRK: combined phototherapeutic keratectomy and photorefractive keratectomy, UDVA: uncorrected distance visual acuity, CDVA: corrected distance visual acuity, SEQ: spherical equivalent

Figure 1 presents four standard graphs reporting the refractive outcome of the two surgical methods in comparison. Regarding efficacy, which is postoperative UDVA in relation to preoperative CDVA, both surgical methods showed equally high results with no loss in Snellen lines (Fig. 1A). Binocular visual acuity (VA) of all patients was 20/20 (Snellen acuity). Similar results can be shown in terms of safety, which describes the postoperative CDVA in relation to preoperative CDVA (Fig. 1B). Therefore, the safety and efficacy indices (SI and EI) were 1.0 for both treatment groups. It is important to note that these visual results were obtained binocular for a more realistic examination of the visual outcome. To calculate predictability, the attempted SE is set in relation to the achieved SE. Here, 100% of eyes in Group A and 95.7% in Group B achieved the attempted SE target within a range of ± 0.50 D (Fig. 1C). There were no differences in predictability between the groups as shown by the Fisher's exact test *(p* = 0.245). The accuracy, indicating the proportion of eyes with a SE within ± 0.50 *D* of the target, is presented in Fig. 1D. All eyes were within this target range, except 4% of eyes in the PTK−PRK group, which were between −1.00 and −0.50 *D*, postoperatively.

## Discussion

PRK is still highly valued today, either as a first treatment for eyes with thin corneas, high refractive error, or as a retreatment after a previous refractive intervention [13]. Therefore, studies investigating different PRK methods are still important. Our data show high efficacy and safety indices for both treatment modalities, *t*PRK and PTK−PRK, without significant differences.

Previous studies have already provided numerous proofs of the high efficacy and safety of the *t*PRK method. Alasmari et al. demonstrated a good refractive outcome with *t*PRK in mild myopia correction [14]. Furthermore, two meta-analyses underline these promising results [7, 15] in addition to several comparative studies [16–18].

Regarding the comparison of the two laser ablation methods, two- and one-step PRK, there seems to be no significant difference in refractive outcomes expressed in the standard parameters of efficacy, safety, and predictability [19]. However, in highly myopic eyes (> −6.00 D), *t*PRK appears to be more effective than the conventional PRK methods [20]. One disadvantage of conventional PRK is the uneven dehydration of the stroma caused either by mechanical- or alcohol-assisted debridement of the corneal epithelium [15]. In contrast, *t*PRK allows for a more consistent corneal ablation, resulting not only in stable refractive outcomes, but also in less pain and haze formation over time [21]. Controversial results may be caused by the different surgical regimens in terms of MMC use or the follow-up period [3].

Laser-assisted epithelial removal, as used in PTK, is a less traumatic technique which provides a smoother ablation profile by reducing the postoperative fibroblast hyperplasia and increasing the epithelial adherence [22, 23]. Published data on the novel PRK method we studied, which consists of PTK treatment followed by PRK, are very scarce. The few available studies have been published on combined PTK−PRK treatment for myopic eyes with corneal scars or other opacities [24]. In addition, this combination is also used to correct complications from previous refractive treatments [25]. This is the first study comparing the *t*PRK technique using the Amaris750 with PTK−PRK performed with MEL90. Tangmonkongvoragul et al. demonstrated good refractive results of the PTK−PRK method for treating mild myopia with the EX500 excimer laser [8]. A recently published study also confirmed the efficacy of PTK−PRK surgery compared to *t*PRK treatment [9]. These results are consistent with our findings. This two-step procedure has some similarities with the *t*PRK, as it is a non-contact laser treatment. Nevertheless, Abdel−Radi et al. demonstrated a faster recovery time in *t*PRK eyes compared with PTK−PRK patients, which they attributed to a shorter surgical time and thus more precise alignment between the epithelial and stromal ablation with respect to their contraction. They concluded that *t*PRK surgery is superior to PTK−PRK regarding visual outcome. The faster recovery of this single-step technique is known from previous studies, which compared *t*PRK to mechanical two-step PRK [16]. Regarding HOA, we found no significant differences between the two groups. However, it is worth mentioning that an aspheric PTK theoretically produces different aberrations than a PTK-based epithelial ablation without aspheric correction, where less energy reaches the peripheral cornea. This may have clinical relevance under scotopic lighting conditions.

The use of MMC in refractive surgery is frequently discussed. While some authors promise stable results without MMC [26], others recommend its use to avoid postoperative complications such as haze and scarring [27]. Some also suggest the use of MMC after PTK treatment [28]. We have used MMC for all our treatments with no postoperative complications in the follow-up period, which is in line with previous works. Abdel−Radi et al. reported minimal haze formation (grade 1) even after MMC application [9]. However, they used a different laser platform, which makes comparison difficult.

A limitation of our study is the short follow-up period, which may have led us to overlook potential postoperative complications. However, from our experience, we know that the use of MMC prevents haze formation in almost all PRK cases, which can be one of the biggest issues after PRK. Further limitations may be the small group size and the lack of comparison data of other study groups using the same laser platforms. Additionally, due to differences in equipment availability at the respective practice locations, different devices were utilized for topographic measurements in the two groups, potentially introducing a bias. However, we made concerted efforts to alleviate this potential influence by ensuring consistent optical zone settings across all employed devices.

Finally, our results show that the visual outcomes of PTK−PRK treatment are not significantly different from those of the *t*PRK method. Our aim of this analysis is to provide the refractive surgeon with evidence for an individual decision before PRK treatment. Two-stage PTK−PRK may be a suitable option for surgeons using the MEL90 excimer laser for the correction of myopia and astigmatism. Further investigations with larger populations and longer follow-up are needed to proof the quality of the PTK−PRK method.
